# NPY1R is a novel peripheral blood marker predictive of metastasis and prognosis in breast cancer patients

**DOI:** 10.3892/ol.2014.2721

**Published:** 2014-11-20

**Authors:** LEI LIU, QIAN XU, LUYANG CHENG, CHUNHU MA, LIJUN XIAO, DAWEI XU, YAXIAN GAO, JIANPING WANG, HONGRU SONG

**Affiliations:** 1 Department of Immunology, Basic Medical Institute, Chengde Medical College, Chengde, Hebei 067000, P.R. China; 2 Department of Central Laboratory, Basic Medical Institute, Chengde Medical College, Chengde, Hebei 067000, P.R. China; 3 Clinical Skills Center, Chengde Medical College, Chengde, Hebei 067000, P.R. China

**Keywords:** breast cancer, neuropeptide Y receptor Y1, tumor marker, circulating cancer cells

## Abstract

The aim of the current study was to evaluate a novel tumor marker, neuropeptide Y receptor Y1 (NPY1R), for the detection of circulating cancer cells and to investigate its clinical significance in breast cancer patients. The Digital Gene Expression Displayer tool of the Cancer Genome Anatomy Project was used to identify the marker gene NPY1R, which is able to detect circulating cancer cells. Nested quantitative polymerase chain reaction was performed to correlate the NPY1R expression levels with the clinicopathological features of 142 breast cancer patients. A follow-up study of 131 of the breast cancer patients was conducted for 38 months. Compared with the 60 normal control individuals, NPY1R was highly expressed in the cancer patients (P<0.01). These high levels of NPY1R expression were positively correlated with the clinical stage and lymph node metastasis status of the disease, as well as with the status of the estrogen and progesterone receptors (P<0.05). Breast cancer patients with circulating cancer cells that expressed NPY1R exhibited shorter tumor-specific survival when compared with those with no NPY1R expression (P<0.01). Additionally, the mortality rate was associated with HER2 expression in the NPY1R positive and negative groups. These results indicate that NPY1R may serve as a useful marker to predict breast cancer metastasis and to evaluate the prognosis of breast cancer patients.

## Introduction

Breast cancer is the second most frequent type of cancer in the world and is by far the most common malignant disease in female individuals (1). Due to advancements in methods for earlier diagnosis and adjuvant therapy, the prognosis of patients with breast cancer has improved in recent years, however, ~12% of breast cancer patients succumb to the disease within the first five years (2). The most recent statistics for China demonstrate that the mortality rate of breast cancer increased by 10% between 2012 and 2013, and increased by 30.5% between 2007 and 2013. Despite the application of the American Joint Committee on Cancer tumor-node-metastasis system for staging and prognosis, ≤30% of lymph node-negative patients ultimately develop recurrent disease (3). This is possibly due to occult metastatic cells, which are undetectable by currently employed methodology, that have spread via the lymphatic or hematogenous systems. Hence, it is clinically important that disseminated tumor cells are detected using effective markers to supplement the staging method, prediction of metastasis and prognosis in breast cancer.

To identify circulating markers for the detection of disseminated tumor cells in breast cancer patients, the current study used *in silico* analysis of the National Cancer Institute Cancer Genome Anatomy Project database (http://cgap.nci.nih.gov/cgap.html). NPY1R, a novel peripheral blood marker, was determined to exhibit the largest differential expression ratios. Neuropeptide Y (NPY) is the most abundant neuropeptide in the mammalian brain and modulates various mechanisms, such as appetite, anxiety, circadian rhythm, memory and blood pressure (4). The effect of NPY may be mediated by a number of NPY receptor subtypes, termed Y1R-6R, of which upregulation of Y1R and Y2R has been reported in numerous types of human carcinoma, including breast cancer, adrenal tumors, renal cell carcinoma and ovarian cancer. This activation of Y1R and Y2R by NPY resulted in tumor cell proliferation, angiogenesis and metastasis (5).

In the present study, the correlation between NPY1R expression and various clinicopathological features of breast cancer patients was analyzed. Therefore, it was proposed that NPY1R may serve as a useful marker to predict cancer metastasis and to evaluate the prognosis of breast cancer patients.

## Patients and methods

### Patients and samples

The present study was conducted on 142 blood samples provided by breast cancer patients, who were histopathologically and clinically diagnosed with breast cancer at the Affiliated Hospital of Chengde Medical College Cancer Center (Chengde, China) between November 2008 and December 2011. The patient age range was 21–82 years, with a mean age of 52 years. In addition, 60 healthy female volunteers (normal group) were enrolled (median age, 49 years; range 22–76 years). No patients received antihormonal treatment, chemotherapy or radiotherapy prior to surgery, and all data, including age, pathological type, tumor size, lymph node metastasis, clinical stage according to the American Joint Committee on Cancer (6), estrogen receptor (ER) status, progesterone receptor (PgR) status, human epidermal growth factor receptor 2 (HER2) score, according to the American Society of Clinical Oncology/College of American Pathologists guidelines (7), and recurrence were obtained from the clinical and pathological records. All participants provided written informed consent and this study was approved by the ethics committee of Chengde Medical College (Chengde, China).

Peripheral blood samples were acquired from superficial veins on the opposite side to the breast cancer using standard percutaneous venipuncture and collected into two citrate sodium-containing tubes; the first 1 ml of blood was collected in the first tube and the subsequent 5 ml was collected in the second tube. The blood sample in the first tube, which may have been contaminated with epithelial cells picked up by the needle when it pierced the skin, was discarded; however, the blood in the second tube was loaded onto a Ficoll-Hypaque layer (Gibco BRL, Carlsbad, CA, USA). Following density gradient centrifugation, the peripheral blood mononuclear cell pellet was collected, washed twice with sterile phosphate buffer solution, snap frozen and stored at −80°C until RNA extraction.

### Identification of candidate marker gene

The complementary DNA (cDNA) Digital Gene Expression Displayer developed by the Cancer Genome Anatomy Project is an expressed sequence tag database, which contains vast amounts information generated from cancer cell lines. Thus, the cDNA Digital Gene Expression Displayer was used to identify genes that were differentially expressed in breast cancer cells and leukocytes. The P-value filter was set at 0.01 and the differentially expressed genes were ranked according to sequence odds ratio (OR). The gene with the highest sequence OR was selected as the candidate marker gene to undergo subsequent quantitative polymerase chain reaction (qPCR) assays.

### RNA preparation and cDNA synthesis

Total RNA was extracted from the blood samples using TRIzol reagent (Invitrogen Life Technologies, Carlsbad, CA, USA) according to the manufacturer’s instructions, treated with DNase I (Promega Corporation, Madison, WI, USA) and quantified using ultraviolet spectrophotometry (UV2000; LabTech, Beijing, China). Furthermore, cDNA was synthesized from 2 μg total RNA using the Advantage™ Reverse Transcriptase-for-PCR kit (Clontech Laboratories, Inc., Mountainview, CA, USA). The integrity of RNA samples and the accuracy of the cDNA synthesis were verified by performing amplification of GAPDH in a standard PCR reaction.

### Nested qPCR assay

A highly sensitive nested qPCR is required to detect just a few circulating cancer cells. The first round of nested qPCR was performed using 1 μl cDNA (dilution, 1:20) with a PCR mixture (Beijing Tian Wei Yaida Technology Co., Ltd., Beijing, China) containing 0.2 μmol/l outer primers for NYP1R (forward, 5′-TATACCACTCTTCTCTTGGTGCTG-3′ and reverse, 5′-CTGGAAGTTTTTGTTCAGGAACCCA-3′), 0.2 mM deoxynucleotide triphosphate, 50 mM Tris-HCl, 10 mM KCl, 5 mM (NH_4_)_2_SO_4_, 2 mM MgCl_2_ and 0.75 U Taq polymerase, to a total volume of 25 μl. The PCR conditions were as follows: 35 cycles at 94°C for 20 sec, 62°C for 20 sec and 72°C for 40 sec, followed by a final extension at 72°C for 10 min.

For the second round of nested qPCR amplification, the reaction mixture contained 2 μl of the first round PCR product, 0.25 μmol/l inner primers for NYP1R (forward, 5′-ATCTGCCCTTGGCCATGAT-3′ and reverse, 5′-AGGCCAGGTTTCCAGAGACA-3′) and SYBR^®^ Green PCR master mix (Applied Biosystems, Warrington, UK), to a total volume of 20 μl. The qPCR assays were performed using an ABI PRISM^®^ 7000 sequence detection system (Applied Biosystems Life Technologies, Foster City, CA, USA) under the following conditions: 94°C for 4 min followed by 40 cycles at 94°C for 15 sec, 58°C for 30 sec and 72°C for 35 sec. All reactions were performed in triplicate and GAPDH mRNA (forward, 5′-ACCACAGTCCATGCCATC-3′ and reverse, 5′-TCCACCACCCTGTTGCTGTA-3′; Shanghai Shenggong Co., Ltd., Shanghai, China), was used as the internal control. The relative quantity of mRNA, normalized against the GAPDH mRNA, was expressed as in terms of cycle threshold (Ct) using the following equations: ΔCt^NPY1R^ = Ct^NPY1R^ − Ct^GAPDH^; ΔΔCt = ΔCt^tumor^ − mean of ΔCt^normal^ If the fluorescence signal was undetected after 40 cycles, the Ct value was defined as the maximum cycle number of 40 for analysis convenience. Furthermore, the differential expression ratio of the candidate marker gene (Q) was calculated using the following equation: Q = 2^−ΔΔCt^. To determine the marker positivity in the present study, receiver operating characteristic (ROC) curves were plotted according to the −ΔCt value in the breast cancer and normal control groups.

### Follow-up

A follow-up study was conducted on 131 breast cancer patients by a telephone interview between November 2008 and December 2011, with additional verification of clinical records. Chest X-rays and mammographies were examined biannually, and liver ultrasound and bone scans were examined annually. A total of 11 patients were lost to follow-up.

### Statistical analysis

Statistical analyses were performed using the Student’s t-test. Survival distributions were estimated using Kaplan-Meier analysis and the log-rank test was performed to assess the statistical significance of differences between the NPY1R-positive and -negative groups. Furthermore, the Mantel-Haenszel method was used to calculate χ^2^ in the stratified correlation analysis between HER2 expression and patient survival rate. Statistical analyses were conducted using SPSS version 17.0 software (SPSS, Inc., Chicago, IL, USA). P<0.05 indicated a statistically significant difference.

## Results

### Identification of the marker gene NPY1R for detecting circulating breast cancer cells

The *in silico* Digital Gene Expression Displayer program search of the National Cancer Institute Cancer Genome Anatomy Project database identified 30,460 sequences in four breast cancer cDNA libraries and 21,036 sequences in five leukocyte cDNA libraries with a P-value filter set at 0.01. Of these, 23 overexpressed genes with a sequence OR of >16 were yielded from the breast cancer and the leukocyte cDNA libraries. NPY1R exhibited the largest differential expression ratios; therefore, nested qPCR of the peripheral blood samples using NPY1R primers was performed. Positive NPY1R gene expression was identified in 5/10 breast cancer patient samples; however, 0/10 normal controls appeared to express NPY1R.

### Expression of NPY1R in the peripheral blood of breast cancer patients

**Figure 1:**
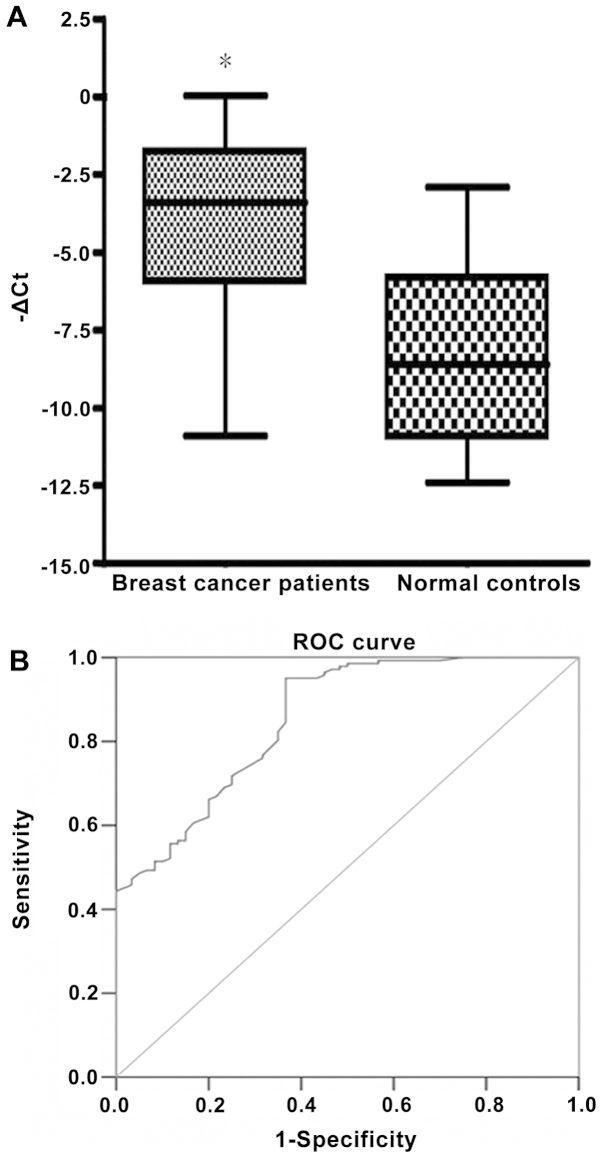
(A) Expression levels of the neuropeptide Y receptor Y1 (NPY1R) gene in the peripheral blood of breast cancer patients compared with normal controls. ^*^P<0.01. (B) Receiver operating characteristic (ROC) curve of the NPY1R marker gene. Area under the curve, 0.855.

Nested qPCR was performed to determine the expression level of NPY1R in the peripheral blood of 142 clinical samples obtained from breast cancer patients. The −ΔCt value, which represents the relative quantity of NYP1R mRNA, was significantly higher in the cancerous samples compared with the corresponding normal control samples (−3.93±2.5 vs. −8.21±2.9; P<0.01; Q, 55.54±27.3; Fig. 1A). The threshold of −ΔCt was set at a conservative value of −2.75; this threshold corresponds to 100% specificity (i.e., no normal control samples were positive) as determined by ROC curve analysis (Fig. 1B). Using this clinical threshold for marker positivity, it was observed that the positive detection rate of circulating cancer cells in 142 breast cancer patients was 44.4% (63/142) for the NPY1R gene.

### Relative expression of NPY1R and patient characteristics

The association between clinicopathological variables and NPY1R transcript expression in the peripheral blood of breast cancer patients was analyzed. High expression levels of NPY1R correlated with the progression of clinical stages (P<0.001). Furthermore, statistical analysis was performed to determine that the HER2 score was significantly higher in the high NPY1R expression group compared with the low NPY1R expression group (P=0.001), the relative NPY1R expression level was significantly higher in ER-positive compared with ER-negative patients (P=0.001), and the relative NPY1R expression level was significantly higher in PgR positive compared with the PgR-negative patients (P=0.037). Of note, high NPY1R expression levels were detected in the lymph node metastasis group, which highlights the value of NPY1R as a predictive peripheral blood marker of lymph node metastasis in breast cancer. However, no statistically significant association was identified between marker detection and tumor size, pathology type or patient age (P>0.05; Table I).

### Association between NPY1R expression and disease progression

**Figure 2:**
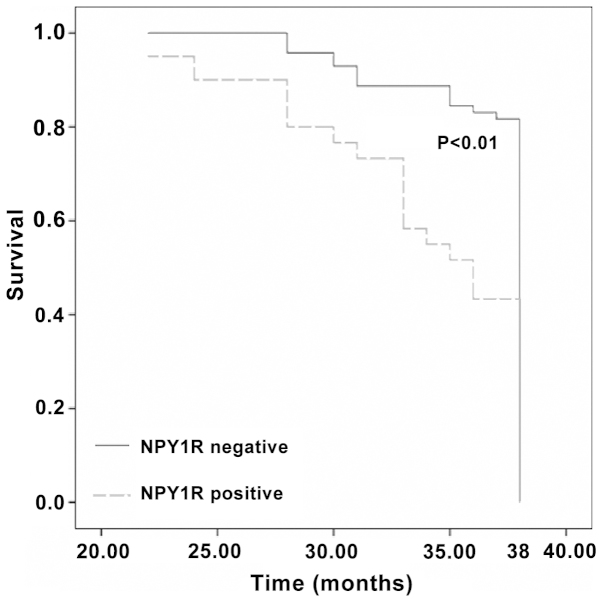
Kaplan-Meier survival analysis of breast cancer patients grouped according to neuropeptide Y receptor Y1 status (NPY1R) in the peripheral blood.

To investigate the association between the detection of circulating tumor cells and the clinical outcome of breast cancer patients, a follow-up study was performed for 38 months in 131 patients following surgical removal of the tumor mass. The survival rate was 61.1% (80/131), of which 46 patients displayed no recurrence. The breast cancer patients with NPY1R-positive circulating cancer cells exhibited shorter tumor-specific survival compared with individuals with absent NPY1R expression (P<0.01; Fig. 2). In addition, the 38-month actuarial overall survival rates were 43.3% (26/60) and 76.1% (54/71) in NPY1R-positive and -negative patients, respectively.

### Association between HER2 expression and patient survival rate in NPY1R-positive and -negative groups

Stratified correlation analysis was performed between HER2 expression and patient survival rate in the NPY1R-positive and -negative groups: NPY1R*-*positive group, χ^2^=4.85 and OR=3.27 [95% confidence interval (CI), 1.14–9.38]; and NPY1R*-*negative group, χ^2^=14.73 and OR=11.08 (95% CI, 3.25–37.77) (Table II). These data indicate that mortality rate is associated with HER2 expression in NPY1R-positive and -negative groups. Furthermore, use of the Mantel-Haenszel method (χ^2^=11.48; P<0.01) and adjusted Mantel-Haenszel method [OR, 4.29 (95% CI, 1.85–9.95)] indicate that HER2 expression is associated with patient survival rate and, therefore, is one of the important risk factors of mortality rate in breast cancer.

## Discussion

The detection of circulating cancer cells holds promise as a powerful tool for cancer diagnosis and disease monitoring (8). However, conventional diagnostic methods, such as imaging and serum marker detection assays, are unable to detect circulating tumor cells as they exist in such small numbers. To overcome this problem, the present study used nested qPCR; a sensitive method that is capable of detecting one breast cancer cell in 10^7^ cells (9). Although various studies have demonstrated that qPCR-based tumor cell detection assays yield higher sensitivity compared with conventional methods, qPCR-based assays for breast cancer have been limited by the availability of molecular markers. Therefore, the present study employed *in silico* analysis to identify marker genes for the detection of circulating tumor cells. The National Cancer Institute Cancer Genome Anatomy Project database and the Digital Gene Expression Displayer program were useful tools for identifying genes that were expressed in the two pools of samples; this also applies to subsequent experiments, providing that a sufficient number of expressed sequence tag libraries for the tissue of interest are archived in the database (10). Differentially expressed genes identified using the abovementioned method may be developed into marker genes for diagnostic or prognostic application by performing experimental verification procedures, such as qPCR. In the present study, nested qPCF was used to identify NPY1R as a novel marker of circulating cancer cells in breast cancer patients; favorable markers are characterized by a high level of expression in breast cancer tissues but no or low expression in the peripheral blood cells of healthy patients (11).

To date, NPY is the most abundant neuropeptide reported in the mammalian brain. In the periphery, NPY is co-stored and co-released with norepinephrine in the sympathetic nerve endings (12). NPY exerts potent biological effects on numerous target areas in the brain and in the periphery. NPY is important in the regulation of the cardiovascular system, lung function, feeding behavior, anxiogenesis, and the release of hypothalamic and pituitary hormones. Kiyokawa *et al* (13) studied the interaction between estrogen, NPY and its receptors, and identified the concerted action of estrogen and progesterone on increased NPY level, as well as the associated increase in luteinizing hormone release (13).

Recent studies have indicated that NPY and its receptors are associated with human cancer. Körner *et al* (14) reported that the Ewing’s sarcoma family of tumors and synovial sarcomas expressed the NPY receptor subtype Y1 at a high incidence rate (84 and 40%, respectively) and density (mean, 5,314 and 7,497 disintegrations/min/mg tissue). Furthermore, a different study identified that numerous types of sarcoma expressed Y1 on intratumoral blood vessels (15). Furthermore, data from a study conducted by Ruscica *et al* (16) indicated that NPY may directly regulate prostate cancer cell growth via its receptor. This regulation appeared to be associated with the kinetics of mitogen-activated protein kinase activation (i.e., long-lasting versus transient) and to the clone-specific involvement of other intracellular signals. The findings indicated that NPY-associated mechanisms may be relevant in the progression of prostate cancer at androgen-dependent and -independent stages of the disease. In addition, a different study proposed a role of NPY in adrenal cortical tumors as well as a Y1R-mediated physiological role in the adrenal gland associated with strong NPY innervation of the cortex (17).

The Y1R was the first NPY receptor subtype to be cloned and characterized. A previous study determined that healthy breast tissue expresses the Y2R subtype, whereas 85% of human breast carcinoma tissue expresses the Y1R subtype (18). The high incidence of the Y1R subtype expression in human breast carcinoma indicates that Y1R may be important in the pathophysiology of breast malignancy; however, the factors responsible for the high incidence of Y1R expression remain unclear. Amlal *et al* (19) hypothesized that the upregulated expression of Y1R was induced by the activation of the estrogen signaling pathway. The effect of estrogen on Y1R mRNA expression and the estrogen signaling pathway were detected *in vitro*. The effect on the estrogen signaling pathway was to induce cell proliferation in breast cancer. It was suggested that the expression of the Y1R gene was increased in response to estrogen treatment by using the MCF-7 cell line, an estrogen receptor-positive human breast cancer cell line, which has been demonstrated to express high-affinity NPY receptors. In addition, a potential mechanism of NPY-inhibited forskolin-stimulated adenosine 3′5′-cyclic monophosphate accumulation and mobilized intracellular Ca^2+^ was proposed in MCF-7 cells. Furthermore, the previous study treated rats with estrogen and examined the upregulation of Y1R mRNA in the hypothalamus by competitive reverse transcription-PCR. These results indicate that estrogen exerts an important effect on the upregulation of the Y1R, which in turn promotes estrogen-induced proliferation in breast cancer cells. An interaction between estrogen, NPY and its receptors has been proposed to explain the concerted action of estrogen and progesterone on increased NPY expression levels and an associated increase in luteinizing hormone release (20). In the present report, it was identified that expression of the marker gene NPY1R in peripheral blood correlated with ER and PgR expression; the expression level of NPY1R was significantly higher in the ER- and PgR-positive groups compared with the negative group. The results indicate that NPY1R may be involved in the activation of the estrogen and progesterone signaling pathway in breast carcinoma.

In the present study, the correlation between HER2 expression and patient outcome was investigated by performing stratified analysis. The results indicated that HER2 expression was associated with patient survival rate and, thus, was one of the important risk factors of mortality in breast cancer. HER2 is overexpressed in 20–30% of breast cancer patients; therefore, recent studies have focused on investigating the role of HER2 as a prognostic indicator to predict poor clinical outcome in breast cancer patients (21). The application of radioimmunohistochemical methods demonstrated that 85% of 296 breast tumor samples overexpressed HER2. Of these, 23% expressed HER2 at 45–480 times greater than the normal level; this high overexpression was associated with a poor clinical outcome, therefore the serum HER2 expression level may be useful to predict a poor clinical outcome in patients with HER2-positive breast cancer. Furthermore, univariate analysis demonstrated that tumor grade, necrosis, lymphovascular invasion and hormone receptor negativity were significantly associated with HER-2/neu overexpression. The upregulation of NPY1R appears to be specific to ER^+^ breast cancer patients and thus, a combination of multiple markers, including NPY1R, may be required to improve the sensitivity and specificity for the detection of circulating breast cancer cells. Future studies, which investigate the cellular mechanisms underlying the role of NPY1R in breast cancer are required, as this may lead to the development of drugs to target NPY1R for breast cancer treatment.

## Figures and Tables

**Table I:** Association between the expression level of the NPY1R gene in the peripheral blood of breast cancer patients (n=142) and patient clinicopathological features.

Clinicalpathological feature	Patients, n	Relative NPY1R expression, −ΔCt (mean ± standard deviation)	P-value
Age, years			
<50	56	−2.51±0.23	
≥50	86	−2.23±1.07	0.350
Pathology			
Invasive ductal carcinoma	98	−2.25±0.85	
Simple cancer	7	−2.42±1.27	
Eczematous cancer	5	−2.48±1.34	
Medullary carcinoma	19	−2.56±1.22	
Invasive lobular carcinoma	13	−2.51±1.08	0.952
Tumor size, cm			
≤2	75	−2.69±1.41	
>2	67	−2.12±0.48	0.055
Clinical stage			
I – II	89	−3.11±1.62	
III – IV	53	−1.72±0.91	<0.001
Lymph node metastasis			
Yes	84	−2.04±1.39	
No	58	−3.17±1.49	0.001
ER			
+	82	−1.96±1.28	
−	60	−2.88±1.12	0.001
PgR			
+	63	−2.17±1.27	
−	79	−2.80±1.34	0.037
HER2			
+	68	−1.86±0.87	
−	74	−2.96±1.07	0.001

NPY1R, neuropeptide Y receptor Y1; Ct, cycle threshold; ER, estrogen receptor; PgR, progesterone receptor; HER2, human epidermal growth factor receptor 2.

**Table II:** Stratified correlation analysis between HER2 expression and patient survival rate in NPY1R-positive and -negative groups, as determined by follow-up (n=131).

	HER2^+^			HER2^−^		
		
Patients, n	NPY1R^+^	NPY1R^−^	Total	NPY1R^+^	NPY1R^−^	Total
Mortalities	24	14	38	10	3	13
Survivors	11	16	27	15	38	53
Total	35	30	65	25	41	66

HER2, human epidermal growth factor receptor 2; NPY1R, neuropeptide Y receptor Y1.

## References

[1] Suh MA, Atashili J, Fuh EA, Eta VA (2012). Breast self-examination and breast cancer awareness in women in developing countries: a survey of women in Buea, Cameroon. BMC Res Notes.

[2] Yilmaz YE, Lawless JF, Andrulis IL, Bull SB (2013). Insights from mixture cure modeling of molecular markers for prognosis in breast cancer. J Clin Oncol.

[3] Park Y, Chang M, Lee S (2009). Heterogeneity of triple negative breast cancer (TNBC): TNBC might be divided into two or more subgroups by clinicopathologic findings. Cancer Res.

[4] Kohno D, Yada T (2012). Arcuate NPY neurons sense and integrate peripheral metabolic signals to control feeding. Neuropeptides.

[5] Memminger M, Keller M, Lopuch M (2012). The neuropeptide y y(1) receptor: a diagnostic marker? Expression in mcf-7 breast cancer cells is down-regulated by antiestrogens in vitro and in xenografts. PLoS One.

[6] Edge SB, Compton CC (2010). The American Joint Committee on Cancer: the 7th edition of the AJCC cancer staging manual and the future of TNM. Ann Surg Oncol.

[7] Wolff AC, Hammond ME, Schwartz JN (2007). American Society of Clinical Oncology/College of American Pathologists guideline recommendations for human epidermal growth factor receptor 2 testing in breast cancer. J Clin Oncol.

[8] Rahbari NN, Bork U, Motschall E (2012). Molecular detection of tumor cells in regional lymph nodes is associated with disease recurrence and poor survival in node-negative colorectal cancer: a systematic review and meta-analysis. J Clin Oncol.

[9] Liu L, Liao GQ, He P, Zhu H (2008). Detection of circulating cancer cells in lung cancer patients with a panel of marker genes. Biochem Biophys Res Commun.

[10] Kavak E, Ünlü M, Nistér M, Koman A (2010). Meta-analysis of cancer gene expression signatures reveals new cancer genes, SAGE tags and tumor associated regions of co-regulation. Nucleic Acids Res.

[11] Blackhall F, Peters S, Kerr KM (2012). Biomarkers. Ann Onc.

[12] Higuchi H (2012). Molecular analysis of central feeding regulation by neuropeptide Y (NPY) neurons with NPY receptor small interfering RNAs (siRNAs). Neurochem Int.

[13] Kiyokawa M, Matsuzaki T, Iwasa T (2011). Neuropeptide Y mediates orexin A-mediated suppression of pulsatile gonadotropin-releasing hormone secretion in ovariectomized rats. J Med Invest.

[14] Körner M, Waser B, Reubi JC (2008). High expression of neuropeptide Y1 receptors in ewing sarcoma tumors. Clin Cancer Res.

[15] Lu C, Tilan JU, Everhart L (2011). Dipeptidyl peptidases as survival factors in Ewing sarcoma family of tumors: implications for tumor biology and therapy. J Biol Chem.

[16] Ruscica M, Dozio E, Boghossian S (2006). Activation of the Y1 receptor by neuropeptide Y regulates the growth of prostate cancer cells. Endocrinology.

[17] Körner M, Waser B, Reubi JC (2004). High expression of neuropeptide y receptors in tumors of the human adrenal gland and extra-adrenal paraganglia. Clin Cancer Res.

[18] Reubi JC, Gugger M, Waser B, Schaer JC (2001). Y(1)-mediated effect of neuropeptide Y in cancer: breast carcinomas as targets. Cancer Res.

[19] Amlal H, Faroqui S, Balasubramaniam A, Sheriff S (2006). Estrogen up-regulates neuropeptide Y Y1 receptor expression in a human breast cancer cell line. Cancer Res.

[20] Sheriff S, Ali M, Yahya A (2010). Neuropeptide Y Y5 receptor promotes cell growth through extracellular signal-regulated kinase signaling and cyclic AMP inhibition in a human breast cancer cell line. Mol Cancer Res.

[21] Lennon S, Barton C, Banken L (2009). Utility of serum HER2 extracellular domain assessment in clinical decision making: pooled analysis of four trials of trastuzumab in metastatic breast cancer. J Clin Oncol.

